# Maternal parenting behavioural profiles and developmental outcomes in early years: a latent profile analysis in rural China

**DOI:** 10.7189/jogh.15.04042

**Published:** 2025-05-12

**Authors:** Mengxue Xu, Hongyi Sun, Aihua Liu, Xuhang Zhao, Yuning Zhang, Hongyan Guan

**Affiliations:** 1 Nurturing Care Research and Guidance Center, Child Healthcare Center, Capital Institute of Pediatrics, Beijing, China; 2 Centre for Innovation in Mental Health, School of Psychology, University of Southampton, Southampton, UK; 3 Department of Integrated Early Childhood Development, Capital Institute of Pediatrics, Beijing, China; 4 State Key laboratory of Cognitive Neuroscience and Learning, Beijing Normal University, Beijing, China

## Abstract

**Background:**

Parenting behavioural profiles differ across cultural, economic, and ethnic contexts. China, with one of the largest child populations worldwide, faces the challenge of poor developmental outcomes during early childhood in rural areas. Using a data-driven approach, we aimed to explore distinct parenting profiles, their corresponding developmental outcomes during early childhood, and the associated family risk factors.

**Methods:**

We enrolled children and their caregivers from a national poverty-stricken county in China. We used the Bayley Scales of Infant and Toddler Development – Third Edition to measure their developmental outcomes by cognition, motor, and language, and we assessed their social-emotional development using the Chinese version of Ages and Stages Questionnaire: Social-Emotional, Second Edition. We used latent profile analysis to examine the patterns of parenting behaviour and examined the difference in developmental outcomes and familial risk factors via analysis of variance.

**Results:**

We interviewed 260 children (mean age = 9.62, standard deviation (SD) = 3.76 months; 51.5% female) and their caregivers from a national poverty-stricken county in China. The two-profile solution best fitted the data and indicated two parenting style patterns: low human stimulation (HS) & low social support (SS) group (n = 61, 23.46%) and high HS & high SS group (n = 199, 76.54%). There was a significant difference in children’s social emotional development (*P* = 0.013) and mothers’ depression score (*P* = 0.046) between the two parenting behavioural patterns.

**Conclusions:**

Our study provides evidence on maternal parenting behaviour, associated risks, and child development outcomes in rural China, with significant implications for further high-quality interventions in regions of comparable economic level, particularly in the rural areas of Western China.

Early childhood development (ECD) encompasses the growth and learning of children from conception to age eight [1]. It plays a crucial role in achieving the Sustainable Development Goals (SDGs) in low and middle-income countries [2]. Unfortunately, around half of children under five worldwide are at risk of not reaching their full developmental potential [3]. In China, unequal resource allocation negatively contributes to the persistent discrepancies in ECD [4]. For example, children under five years of age in rural areas are confronted with a risk of cognitive, language, and social-emotional delays at respective percentages of 45%, 46%, and 36% (n = 19 762) [5]. Children living in counties designated as national-level poverty-stricken areas are particularly vulnerable to this risk [6]. Among the key influencing factors of nurturing care quality such as ethnicity, socioeconomic status, and maternal education [7-9], parenting behaviour is one of the most essential and mutable determinants [10,11].

Several widely-used questionnaires exist for measuring parenting behaviours, such as the Knowledge of Infant Development Scale Inventory [12], the Parenting Scale [13], Home Observation for Measurement of the Environment (HOME) [14], and Parenting Child Interaction Scales [15]. They are all focussed on the duration and/or frequency of parenting practices such as reading/storytelling, singing, outdoor time, and play. Compared with self-reported questionnaires, observational evaluation of parent-child interaction is better for measuring the quality of parenting practices, as it removes bias inherent to self-reporting [16]. However, it is unable to widely utilise this time-consuming measuring approach to extensively implement in large-scale population studies or routine clinical visits. Hence, it is important for both research communities and pediatric practitioners to find a way to balance both practicality and precision.

Properly categorising these behaviours provides key insights for guiding interventions and shaping effective decision-making processes. The current literature has a notable limitation: it tends to oversimplify essential subgroups within parenting profiles by focussing only on bivariate associations [17]. Such a confirmatory approach (*i.e.* categorisations of different parenting behaviours based on score intervals) has been widely used in parenting profile research and validated by various empirical studies [18-20]. Nevertheless, this method fails to identify naturally occurring parenting patterns, which may inadvertently coerce certain populations into pre-defined, theoretically-based categories [19].

Culture plays a pivotal role in shaping parenting behaviour. Early research on this topic tended to conclude that authoritative parenting was the most beneficial for child development, and it was thought that the relevant research findings were globally applicable [21,22]. For instance, a study examining British adolescents identified three distinct parenting styles – authoritative, authoritarian, and permissive – and found that family structure significantly influenced these styles [21]. A study based on a USA national cohort reported that the authoritative parenting style is the most conducive to children’s educational outcomes [23]. Nevertheless, the limited research conducted outside Western countries has highlighted that parenting behaviour differs by culture [24], as well as socioeconomic or ethnic background [25]. For instance, a recent investigation has indicated that urban Chinese parents exhibit high levels of engagement in their children’s education and seldom resort to punishment, while among caregivers, the authoritarian parenting style and reduced involvement in educational activities were more prevalent [7]. These differences in parenting practices between countries, both generally and by their SES, highlight the need for more investigation into varied cultural, ethnic, and SES backgrounds, to allow for more effective design of context-specific interventions.

Here we wanted to characterise distinctive maternal parenting behaviour profiles in a representative, resource-limited rural county in China, and to further investigate the familial risk factors that correspond to varying patterns of parenting behaviour.

## METHODS

### Recruitment and procedure

We carried out recruitment and data collection in Fenxi County, Linfen City, Shanxi Province, designated as one of the 680 national-level poverty-stricken counties prior to China’s official declaration of the complete eradication of poverty in 2019 [26]. Gross domestic product per capita in Fenxi County in 2019 was USD 2151 [27], much lower than that of China overall (USD 10 144) [28] and Shanxi Province (USD 2465) [29] in 2021.

All children aged between 4 to 16 months were eligible for inclusion in the study in May 2019. Trained village doctors organised the enrolment and asked primary caregivers to read and sign an informed consent form upon agreeing to participate. The data we report here was the baseline data of the Care Group Intervention [30,31]. During the analysis phase, we excluded children with disabilities, incomplete responses to the questionnaires, and cases where the caregiver was not the biological mother, and randomly selected one child in cases of twins/multiple births. We obtained ethical approval from the Ethical Committee of the Capital Institute of Paediatrics, Beijing, China (SHERLL2018014).

### Measurements

#### Parenting behaviour

Mothers reported their parenting behaviour via the Index of Child Care Environment (ICCE), a self-reported scale with proven consistency with the HOME (r = 0.76; *P* < 0.01) [18], consisting of 13 items across four subscales:

1. Social Stimulation, which evaluates the frequency of positive stimulation outside of caregiver-child dyads (comprising three items, such as ‘Do you often go to the park or play outside with your child during the week?’);
2. Human Stimulation (HS), which measures the frequency of positive stimulation within caregiver-child dyads (comprising five items, *e.g.* ‘How often do you play with your child per week?’);
3. Avoidance of Restriction, which assesses the frequency of harsh parenting (comprising two items, for instance, ‘How many times did you hit or kick your child last week?’);
4. Social Support (SS), which quantifies the assistance received in child rearing form various sources, including spouse, friends or relatives, and professions (comprising three items, for example, ‘Does someone help you take care of your child?’).

Items 1 through 11 are evaluated on a five-point Likert scale, which reflects varying frequencies of occurrence (1 = very little; 2 = 1–2/months; 3 = 1–2/week; 4 = 3–4/week; 5 = almost every day). Items 12 and 13 inquire whether the caregiver has access to assistance or consultation for childcare. These two items are formatted as binary choices. The cumulative score for the subscale is determined by summing the responses to all items. Higher scores indicate superior quality of care within each domain.

#### Infant and toddler developmental outcomes

We considered cognitive, motor, and language development, social-emotional development, and physical development as outcomes in our analysis

Trained graduate students majoring in Psychology or Education conducted the test for cognitive, motor, and language development using the Bayley Scale of Infant and Toddler Development – Third Edition (BSID-III) [32]. The Chinese version has been standardised in children under three years old and has been widely used in studies on China’s poverty-stricken areas [25,31,32]. A higher composite score represents a better developmental level in each domain.

The assessment for social-emotional development was conducted with the Chinese version of the Ages and Stages Questionnaire: Social-Emotional, Second Edition (ASQ: SE-2), a tool known for its good validity when applied to Chinese children [33]. ASQ: SE-2 has three response options for all items: 0 = rarely or never; 5 = sometimes, and 10 = most of the time. Five extra points are added if the item is considered a concern. Higher total scores indicate lower levels of social-emotional development. We adopted the Z-score methodology to evaluate the progress of individuals in social-emotional development across different age groups. This method was crucial due to the fundamental differences between the questions, as well as the varying number of questions at each developmental stage, since these variations could result in misleading and unfair direct comparisons.

Regarding participants’ physical development, trained reviewers measured their height (cm) and weight (kg) during visitation, and we calculated and categorised their body mass index (BMI) according to the World Health Organization BMI-for-age guide [34].

#### Familial risk factors

Mental health plays a crucial role in shaping behavioural development, with maternal depression serving as a representative factor. Mothers who experience symptoms of depression may exhibit reduced interaction with their children and may not respond adequately to or may even ignore their children’s emotional needs. As a result, our study has identified maternal depression as a critical mediating variable. Mothers self-reported their depressive symptoms by completing the 20-item Centre for Epidemiological Survey-Depression Scale (CES-D), which employs a four-point Likert scale for response [35] (*e.g.* ‘I thought my life had been a failure’; 0 = less than 1 day, 1 = 1–2 days, 2 = 3–4 days, 3 = 5–7 days). The total composite score ranges between 0 and 60; a higher score represents more severe depressive symptoms.

In terms of SES, we recognised in our pilot study that annual family income is a highly sensitive indicator which often leads caregivers to hesitate or even refuse to disclose. Consequently, we used the number of household items as a proxy measure to assess the standard of living. Caregivers reported whether their family possessed the following 16 items that could reflect family living quality: bike, motorcycle, motorised bicycle, family car, farm car, tractor/ tricycle, landline/non-smartphone, smartphone, refrigerator, washing machine, electric rice cooker, air conditioner, computer, iPad, tap water installation, and Wi-Fi internet. This led to a composite score ranging from 0–16.

### Statistical analysis

We evaluated the normality of all continuous variables through the Shapiro-Wilk test, summarising data that followed a normal distribution using the mean (x̄) and standard deviation (SD). We otherwise described categorical variables using frequencies and percentages. We utilised three steps to explore the best-fitting model, evaluate group differences, and examine the model:

To explore patterns of parenting behaviour, we conducted latent profile analysis (LPA) using Mplus, version 8.3 (Muthén & Muthén, Los Angeles, CA, USA). This approach employs a more rigorous and robust statistical framework to ascertain the optimal number of classifications, offering a higher degree of objectivity compared to conventional methods such as correlation or regression analysis. We selected the best-fitting model based on its substantive interpretability rather than solely focussing on individual indicators [36]. However, we still did assess some indicators, namely the Akaike information criterion (AIC), Bayesian information criterion (BIC), and sample-adjusted BIC (SABIC), and numbers of individuals in different patterns of parenting behaviour. A statistical model is recognised as optimal when it optimally satisfies the following criteria: classification entropy values approaching 1; statistically significant improvement over nested models, as evidenced by Lo-Mendell-Rubin likelihood ratio test (LMRT) and parametric bootstrapped likelihood ratio test (BLRT) *P*-values <0.05; and moderately.sized latent class memberships that maintain analytical power without compromising subgroup distinction. The analytical code for the LPA conducted using Mplus is available in the **Online Supplementary Document**.

To evaluate the developmental outcomes of infants and toddlers according to different parenting behaviour profiles/patterns, we opted either for analysis of variance or mixed models based on the distribution characteristics of the sample data by Statistical Package for the Social Sciences (SPSS), version 21.0 (IBM SPSS Statistics, Chicago, IL, USA) for building our models. We controlled for a range of variables within the models, including the mothers’ levels of education and depression, the duration of gestation, as well as the children’s gender, birth weight, and birth order.

To examine familial risk factors that distinguish parenting behaviour patterns, we adopted the identical procedure we used in our LPA in SPSS. The controlled-for variables were the mothers’ levels of education and depression, the duration of gestation, as well as the children’s gender, birth weight, and birth order.

## RESULTS

**Figure 1:**
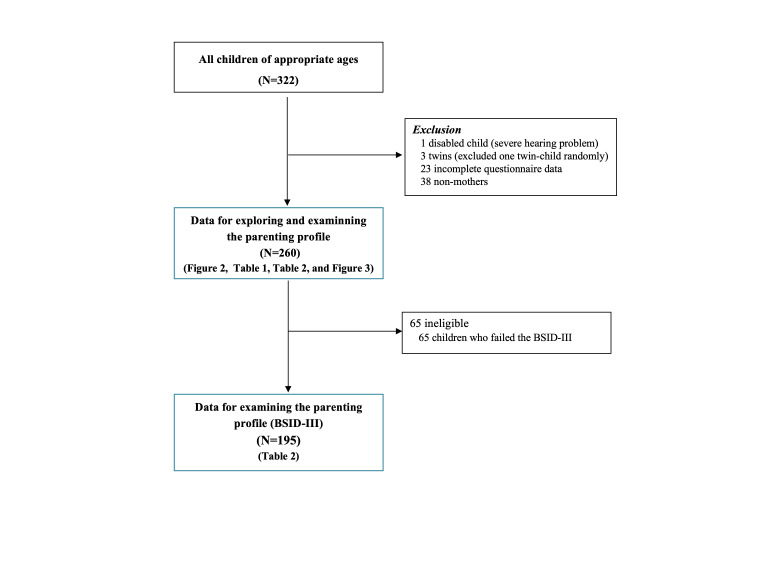
Trial profile.

Although frequent migration between the townships and the nearby urban areas impacted our enrolment, we have included all the children of appropriate age in the entire county. Of 322 caregiver-child dyads that participated in the study, we excluded data for children with physical disabilities (n = 1, severe hearing problem); those who had a twin or sibling who also participated in the same study (n = 3, excluded at random); those who did not provide answers on ICCE (n = 23); and those for whom the participating caregiver was not the mother (n = 38). This left 260 mother-child dyads for our analysis. Among them, 65 children did not fully complete the BSID-III; we therefore included 195 samples for group comparison to examine the familial risk factors that differentiate parenting behavioural patterns (**Figure 1**; Table S1 in the **Online Supplementary Document**).

### Participants’ demographics and descriptive statistics

**Figure 2:**
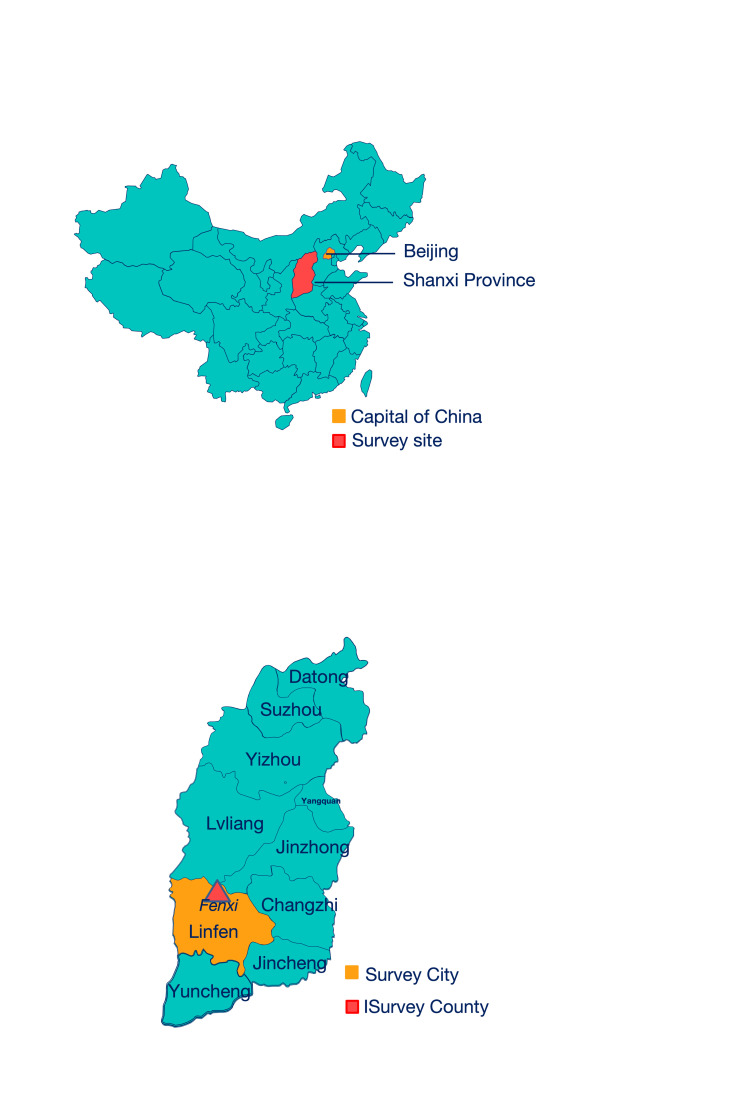
Survey site.

The demographic characteristics and the primary outcome (child cognitive and non-cognitive scores) were normally distributed. There were 134 girls (51.5%) and 126 boys (48.5%), with an average age of 9.62 months (SD = 3.76). Among the mothers, 46.15% had a junior high school or higher level of education. For the ICCE subscale assessments, we recorded a mean score of 19.2 (SD = 3.56) for human stimulation, 7.5 (SD = 3.52) for social stimulation, 6.53 (SD = 0.84) for avoidance of restriction, and 7.53 (SD = 1.53) for social support. Regarding developmental outcomes, children achieved mean composite scores of 101.22 (SD = 14.03) in cognitive development, 98.10 (SD = 12.79) in language development, and 98.93 (SD = 17.34) in motor development. Further, 71.9% of children exhibited typical social-emotional development, while maternal mental health data indicated that 75.4% of mothers showed no signs of depression or suspected depressive symptoms (**Figure 2**; Table S1 in the **Online Supplementary Document**).

### Parenting behavioural profiles

**Figure 3:**
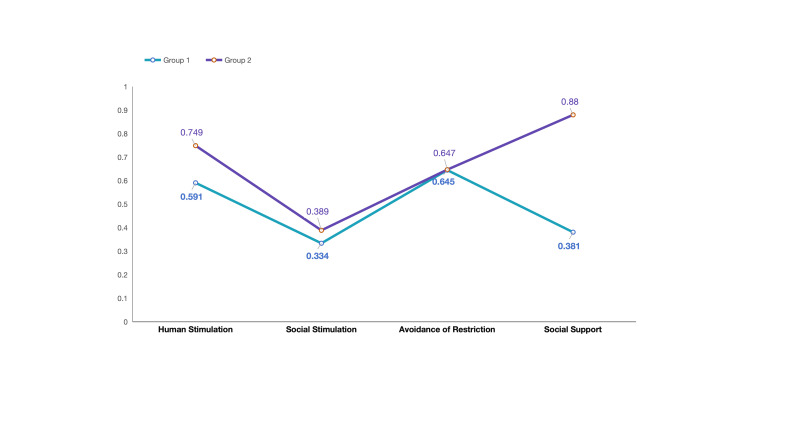
Latent profile model (two groups) based on the ICCE (normalised). Group 1: low human stimulation and social support group (n = 61, 23.46%). Group 2: high human stimulation and social support group (n = 199, 76.54%). H-HS&SS – high human stimulation and social support group, L-HS&SS – low human stimulation and social support group.

Based on the values of AIC, BIC, and SABIC for profile solutions 1–5, the four-profile model showed the best model fit (**Table 1**). However, class membership probability indicated there were only three people in one of the profiles, making it less meaningful and harder to interpret. The three-profile model was abandoned for the same reason, leaving the two-profile model to be the best-fitting model of the data (**Figure 3**). The two-profile model (low human stimulation/social support group and high human stimulation/social support group) differed significantly in human stimulation (x̄ = 16.97, SD = 3.77 *vs.* x̄ = 19.88, SD = 3.20; *P* < 0.001) and social support (x̄ = 5.15, SD = 0.79 *vs.* x̄ = 8.26, SD = 0.77; *P* < 0.001).

**Table 1:** Model fit information of latent profile analysis using ICCE (n = 260)*

Profile model	AIC	BIC	SABIC	Entropy	*P*-LMRT	*P-*BLRT
1	4412.237	4440.722	4415.359			
2	4301.443	4347.732	4306.517	0.882	<0.0001	<0.0001
3	4287.307	4351.400	4294.333	0.922	0.2099	<0.0001
4	4263.519	4345.414	4272.495	0.929	0.4726	<0.0001
5	4256.149	4355.848	4267.077	0.848	0.3970	0.2632

AIC – Akaike information criterion, BIC – Bayesian information criterion, BLRT – parametric bootstrapped likelihood ratio test, LMRT – Lo-Mendell-Rubin likelihood ratio test, SABIC – sample-adjusted BIC

*Three profiles: class 1 (n = 2), class 2 (n = 4), class 3 (n = 195). Four profiles: class 1 (n = 38), class 2 (n = 3), class 3 (n = 159), class 4 (n = 60). Five profiles: class 1 (n = 43), class 2 (n = 1), class 3 (n = 101), class 4 (n = 19), class 5 (n = 96).

Drawing on the items from the HS and SS subscales, high HS/SS represented mothers engaged in frequent interactive activities with their children, such as reading, playing, and storytelling. Additionally, these mothers have ready access to emotional and nurturing support from their spouses, relatives, friends, or professional networks. Conversely, lower scores (Low HS/SS) indicate a reduced frequency of parent-child engagement and diminished support in parenting.

### Group differences in child development

We then compared the low HS/SS group (n = 61, 23.46%) and the high HS/SS group (n = 199, 76.54%) were then compared on all developmental outcomes (**Table 2**). There was a significant difference in social-emotional development, with the high HS/SS group (x̄ = −0.08, SD = 0.90) scoring significantly higher than the low HS/SS group (x̄ = 0.18, SD = 1.49; F-test (195) = 6.255; *P* = 0.013). The two groups did not differ in physical development, meaning in height (F-test (195) = 1.236; *P* = 0.268), weight (F-test (195) = 2.457; *P* = 0.119), and BMI (F-test (195) = 0.343; *P* = 0.559). We also found no significant differences in cognitive (F-test (195) = 0.127; *P* = 0.772), language (F-test (195) = 0.222; *P* = 0.638), or motor (F-test (195) = 0.338; *P* = 0.562) development.

**Table 2:** Group comparison on developmental outcomes and familial risk factors

Dependent variables	Low HS/SS group, (n = 61, 23.46%), x̄ (SD)	High HS/SS group, (n = 199, 76.54%), x̄ (SD)	*P*-value (F-test)
Parenting behaviour (ICCE) (n = 260)			
*Human stimulation*	16.97 (3.77)	19.88 (3.20)	<0.001 (35.54)
*Social stimulation*	7.10 (3.23)	7.62 (3.60)	0.309 (1.038)
*Avoidance of restriction*	6.54 (0.81)	6.52 (0.86)	0.882 (.022)
*SS*	5.15 (0.79)	8.26 (0.77)	<0.001 (758.079)
Developmental indicators (BSID-III) (n = 195)			
*Cognitive score*	101.82 (13.81)	100.96 (14.13)	0.772 (0.127)*
*Language score*	97.36 (11.21)	98.40 (13.24)	0.638 (0.222)*
*Motor score*	100.14 (16.08)	98.41 (17.66)	0.562 (0.338)*
*Social emotional development (ASQ: SE-2) (N = 260)*‡	0.18 (1.49)	-0.08 (0.90)	0.013 (6.255)
Physical developmental outcomes (n = 260**)**			
*Height*	7.82 (5.94)	70.76 (5.60)	0.268 (1.236)
*Weight*	9.54 (1.74)	9.14 (1.42)	0.119 (2.457)
*BMI*	18.39 (1.68)	18.22 (1.69)	0.559 (0.343)
Familial risk factors (n = 260)			
*Maternal depression (CES-D)*	11.45(7.57)	8.74 (7.95)	0.046 (4.047)†
*Household items*	8.68 (1.85)	8.82 (1.77)	0.650 (0.207)†

ASQ: SE-2 – The Ages and Stages Questionnaire: Social-Emotional, Second Edition, BSID-III – Bayley Scale of Infant and Toddler Development – Third Edition, CES-D – Center for Epidemiological Survey-Depression, HS – Human Stimulation, ICCE – Index of Child Care Environment, SS – Social Support, x̄ – mean

*The covariant included child age, gender, birth gestational age, maternal educational level, and socioeconomic status.

†The controlled variables were the mothers' education level, gestation duration, child gender, birth weight, and birth order.

‡ASQ: SE-2 household items included: bike, motorcycle, motorised bicycle, family car, farm car, tractor/ tricycle, landline/non-smartphone, smartphone, refrigerator, washing machine, electric rice cooker, air conditioner, computer, iPad, tap water installation, and Wi-Fi internet.

### Group differences in familial risk factors

The low HS/SS group mothers reported significantly higher depression scores than the high HS/SS group (x̄ = 11.45, SD = 7.57 *vs.* x̄ = 8.74, SD = 7.95; F-test (195) = 4.047; *P* = 0.046). There was no significant difference in household items between the two groups (x̄ = 8.68, SD = 1.85 in the low HS/SS group *vs.* x̄ = 8.82, SD = 1.77 in the high HS/SS group; F-test = 0.650; *P* = 0.207) (**Table 2**).

## DISCUSSION

This is the first study to examine maternal parenting behaviours and their impact on early childhood development from a person-centred perspective in a representative impoverished area in China. Using LPA, we identified variations in parenting behaviours in terms of HS and SS. Children from families with higher levels of HS and SS showed better social-emotional development, while mothers with lower levels of HS and SS exhibited higher levels of depression.

We observed that HS, characterised by dyadic interactions between mothers and infants such as storytelling, play, singing, and shared mealtimes with parents, is particularly prominent within our target population. Research has consistently shown that mothers are often the most perceptive and responsive caregivers, transcending socioeconomic boundaries [37]. According to the social learning theory, individuals, particularly infants and toddlers who lack fully developed logical reasoning skills, tend to acquire new behaviours by observing and mimicking others [38]. During highly frequent daily interactions with their mothers, children provide feedback through a variety of verbal and non-verbal cues, such as laughter, frowning, reaching out, and eye contact, while caregivers finely adjust their behaviour in response. This supports the idea that parent-child interaction has a substantial impact on infants’ social-emotional development [39]. Although this observational study cannot establish causation, our findings are consistent with those of subsequent intervention studies, wherein the total scores achieved by the intervention group were markedly higher than those of the control group, suggesting a potential positive effect of the interventions [30].

SS, which encompasses the assistance and encouragement that mothers receive from their immediate family members and professionals, emerged as a significant differentiating factor between the two groups in our study. Specifically, mothers who lacked nurturing support, particularly from their spouses, were more prone to experiencing multiple stressors. This phenomenon aligns with the predictions of the Family Stress Model [40]. Moreover, SS may serve as a risk factor for variations in mothers’ depression levels [41,42]. A cross-sectional study from Japan reported that mothers who lack SS from partners and others are 7.22 times more likely to suffer from postpartum depression compared to those with extensive SS [43]. Consequently, deterioration in maternal mental health can impair the effectiveness of mother-child interactions, leading to a less positive nurturing environment for infants and toddlers [44,45].

Our sample had comparable scores on both the SS (the quality of infants’ interactions outside the parent-child dyad) and Avoidance of Restriction (the presence of harsh parenting) subscales. We hypothesise that this similarity arises due to the high uniformity in broader environmental contexts, such as the existence of supermarkets and parks within the local area. Indeed, town planning exhibits a notable degree of similarity in Fenxy County to that observed in the rural regions of western China, especially the more poverty-stricken areas. This could be why we observed no significant differences between the groups. In discussing harsh maltreatment, it is important to acknowledge the cultural nuances present in Asian countries. In some contexts, caregivers may gently kick a child’s bottom as a form of affectionate expression, rather than as an act of punishment. In other scenarios, a caregiver might feign disciplinary action by giving a light kick, which is intended to help the child learn appropriate behavioural norms [46]. This method is considered by some to be an effective means of guiding children towards positive behaviour [47]. This might be a reason for the prevalence of maltreatment, namely the Avoidance of Restriction subscale.

There were no significant differences in physical, language, cognitive, and motor development in the two parenting groups. As for the outcomes of physical development, such as height, weight, and BMI, the primary causal factors for such differences typically include genetic predispositions, nutritional intake, and lifestyle habits, including sleep duration and the quality of physical activity [48]. This means that parenting behavioural style alone cannot account for variations in physical development. Regarding other cognitive outcomes (language, cognition, and motor development), they are shaped by a confluence of genetic factors and environmental influence. Empirical research indicates that enhancing cognitive skills necessitates frequent and extended interventions [49,50]. This suggests that, while disparities in parenting behavioural profiles between two groups in our analysis may indeed affect children’s social-emotional growth, they are unlikely to suffice in producing substantial variations in the cognitive capabilities of these children.

China has already carried out a series of poverty alleviation interventions (*e.g.* infrastructure construction, living conditions, and public services) in impoverished areas which have yielded significant benefits [51]. Considerable progress has also been achieved in the realms of children’s health and compulsory education. The national preschool enrolment rate climbed to 89.7% in 2021, marking a 1.6 percentage point increase from the previous year. Notably, efforts to prevent diseases usually prevalent among children have been intensified, with the vaccination coverage rate for the national recommended program exceeding 90% as of 2022 [52]. Nevertheless, it's important to note that children aged 0–3 are primarily raised at home, where parental awareness and actions are key to improving early childhood development. Although the government is attempting to establish nursery institutions, advancing both parenting skills and maternal mental health remains a crucial public concern that still needs attention and further efforts.

### Strength and limitations

We used LPA, a person-centred method, to identify distinct groups of parenting behaviour and to examine the risk factors associated with different parenting behavioural profiles. The two-profile classification (distinguishing between the low and high human stimulation/social support groups) suggests that increased positive nurturing stimulation is significantly associated with enhanced social-emotional development in children. This indicates that future interventions should prioritise enhancing the quality of parent-child interaction, rather than the frequency of activities in a broad sense. For instance, using a video-based intervention can help caregivers learn to identify children’s verbal and non-verbal cues, going beyond just offering age-appropriate play or reading activities [53]. Group-based interventions offer a distinct advantage by presenting a concrete model of learning and interaction that both caregivers and children can emulate [31].

The investigation site, Fenxi County, is located in central China and is representative of a national-level poverty-stricken county. Our rigorous analysis therefore offers valuable insights and serves as a reference for further ECD interventions or policy consideration in similar contexts. However, due to the study’s observational design, we could not establish causal inferences. Moreover, the sample specifically targeted mother-child dyads, which may exhibit distinct nurturing characteristics compared to other caregiver-child dyads. Consequently, we are cautious of generalising our findings, as they should be only considered representative of maternal parenting styles within impoverished communities. The increasingly frequent migrations between townships and nearby urban areas resulted in an unstable living environment [54]. This suggests that interventions should emphasise the maintaining the consistency and coherence of positive parenting practice.

## CONCLUSIONS

According to our findings, HS and SS are at the core of parenting interventions for ECD, rather than Social Stimulation and Avoidance of Restriction, in impoverished areas of China. This highlights the key elements for further high-quality parenting intervention in regions of comparable economic levels, particularly in the rural areas in Western China.

## Additional material


Online Supplementary Document
