# Supplementary material for: Maternal parenting behavioural profiles and developmental outcomes in early years: a latent profile analysis in rural China
Source: J Glob Health. 2025 May 12;15:04042. doi: 10.7189/jogh.15.04042 (PMC12068196; doi:10.7189/jogh.15.04042)
Supplement: Online Supplementary Document [file jogh-15-04042-s001.pdf]

```
title: LPA for parenting style;
data: file is C:\lpa\LPA_DATA.csv;
variable: names = HS SS AR ST;

                 class = c(2);
analysis: Type = mixture;
model: %overall%
Output:TECH1 TECH8 tech11 tech14;

Plot: type = plot3;
          series = HS(1) SS(2) AR(3) ST(4);
Savedata:   File is LPA_2.txt;
                  Save is CPROB;
```

Table S1. Demographic characteristics

Demographic information	N (%) Or Mean (SD)	Range
Child Gender (male)	134 (51.5%)	
Age (months)	9.62 (3.76)	3-18.2
**Parental Education**	(Mother)	(Father)
Primary School or below	10 (3.8%)	8 (3.1%)
Junior High School	110 (42.3%)	139 (53.5%)
Senior High School	90 (34.5%)	76 (29.2%)
higher vocational school	35 (13.5%)	29 (11.2%)
University or above	15 (5.8%)	7 (2.7%)
Unknown	0 (0%)	1 (0.4%)
**Parenting Style (ICCE)**		
Human Stimulation	19.2 (3.56)	5-25
Social Stimulation	7.5 (3.52)	3-15
Avoidance of Restriction	6.53 (.84)	2-9
Social Support	7.53 (1.53)	3-9
**Infant and toddler developmental outcomes**		
Developmental indicators (BSID-III) [a]		
Cognitive Score	101.22 (14.03)	55-130
Language Score	98.10 (12.79)	29-132
Motor Score	98.93 (17.34)	7-139
Social Emotional development (ASQ: SE-2) †		
Normally developed	187 (71.9%)	
Monitoring Zone	73 (28.1%)	
Physical developmental outcomes		
Weight (kg)	9.23 (1.51)	5.25-13.25
Height(cm)	70.86 (5.52)	56.35-87
BMI	18.32 (1.68)	14.52-23.87
**Familial Risk Factors**		
Maternal Depression (CES-D)	9.35 (7.91)	0-48
Normal	196 (75.4%)	0-15
Suspected Depression	36 (13.8%)	16-19
Depression	28(10.8%)	20-48
Household items (Total score) ‡	8.79 (1.78)	0-16

†*The sample size is 195 due to incompleshed assessment.*

‡*Household items is the sum of 16 household appliers that can reflect living quality.*

ICCE: The Index of Child Care Environment; The BSID-III: Bayley Scale of Infant and Toddler Development- III; CES-D: The Center for Epidemiological Survey-Depression; ASQ:SE-2: The Ages and Stages Questionnaire: Social-Emotional, Second Edition.
